# USING COLLECTIVE IMPACT TO ASSESS HOME AND COMMUNITY-BASED SERVICES WORKER TRAINING AND CREDENTIALING

**DOI:** 10.1093/geroni/igad104.3781

**Published:** 2023-12-21

**Authors:** Sandi Lane, Zavera Basrai, Caroline Yoon, Trish Farnham, Kezia Scales, Erin Carson, Nathan Boucher

**Affiliations:** Appalachian State University, Boone, North Carolina, United States; Duke University, Durham, North Carolina, United States; Duke University, Durham, North Carolina, United States; NC Coalition on Aging, Raleigh, North Carolina, United States; PHI, New York City, New York, United States; National Domestic Workers Alliance, Durham, North Carolina, United States; Veterans Health Administration, Durham, North Carolina, United States

## Abstract

Older adults and people living with disabilities receive home- and community-based services (HCBS) from approximately 120,000 often poorly paid and inadequately supported direct care workers in North Carolina (NC). The demand for these workers is increasing rapidly, with NC projected to add almost 23,000 new direct care jobs from 2020 to 2030. With the goal of optimizing the workforce pipeline to prepare for the high need for HCBS across NC, we conducted a Medicaid-funded landscape analysis of direct care training/credentialing using Collective Impact (CI). CI is used for diverse collaborations through forming a common agenda, shared measurement, mutually reinforcing activities, continuous communication, and “backbone” support (dedicated coordination team). Engaging with lived experiences is crucial in CI as it leads to sustainable decision-making informed by those affected by policy change. With input from long-term services and support agencies, licensing bodies, advocates, and those with lived experience, we characterized NC HCBS training/credentialing landscape, generating a first-of-its-kind comprehensive crosswalk resource for the state. Findings reveal the complexity of training/requirements, limited portability, lack of flexibility, and a need for more person-centered and relational core competencies. In addition, funding and incentives for additional training are lacking, which limits opportunities for professional development and career advancement. CI is an effective method to analyze direct care worker training/credentialing, it allowing researchers to harness the wisdom of community partners and incorporate lived experiences of the people most impacted by potential policy changes - workers, HCBS recipients, and families.

